# Essential Oils against *Candida auris*—A Promising Approach for Antifungal Activity

**DOI:** 10.3390/antibiotics13060568

**Published:** 2024-06-19

**Authors:** Adam Kowalczyk

**Affiliations:** Department of Pharmacognosy and Herbal Medicines, Faculty of Pharmacy, Wroclaw Medical University, 50-556 Wrocław, Poland; adam.kowalczyk@umw.edu.pl

**Keywords:** *Candida auris*, essential oils, antifungal agent

## Abstract

The emergence of *Candida auris* as a multidrug-resistant fungal pathogen represents a significant global health challenge, especially given the growing issue of antifungal drug resistance. This review aims to illuminate the potential of essential oils (EOs), which are volatile plant secretions containing complex mixtures of chemicals, as alternative antifungal agents to combat *C. auris*, thus combining traditional insights with contemporary scientific findings to address this critical health issue. A systematic literature review was conducted using the PubMed, Scopus, and Web of Science databases from 2019 to 2024, and using the Reporting Items for Systematic Reviews and Meta-Analysis (PRISMA) protocol to identify relevant studies on the antifungal efficacy of EOs or their components against *C. auris*. Of the 90 articles identified, 16 were selected for detailed review. The findings highlight the diverse mechanisms of action of EOs and their components, such as disrupting fungal cell membranes, inducing the production of reactive oxygen species (ROS), and impeding biofilm formation, suggesting that some of them may be as effective as, or better than, traditional antifungal drugs while potentially limiting the development of resistance. However, issues such as variability in the composition of EOs and a paucity of clinical trials have been identified as significant obstacles. In conclusion, EOs and their active ingredients are emerging as viable candidates for creating effective treatments for *C. auris*, underscoring their importance as alternative or complementary antifungal agents in the face of increasing drug resistance. The call for future research underscores the need for clinical trials and standardization to unlock the full antifungal potential of EOs against *C. auris*.

## 1. Introduction

In recent times, the global healthcare system has experienced an amplified burden due to the emergence of multidrug-resistant pathogens. Among these, *C. auris* is particularly notable due to its alarming resistance patterns to common antifungal agents used to treat these infections and its associated high mortality rates. First detected in 2009, *C. auris* has demonstrated rapid global dissemination and has been identified in 35 countries [1]. The yeast identification methods used by laboratories often misidentify *C. auris* as other yeasts, which makes the detection and control of this pathogen particularly challenging. *C. auris* transmission occurs in healthcare settings, even in those with rigorous infection prevention and control measures in place. The Centers for Disease Control and Prevention (CDC) categorizes this pathogen as an urgent threat in antimicrobial resistance, highlighting the critical need for innovative treatment strategies [2,3].

*C. auris* is distinct in its ability to efficiently spread from person to person, unlike other *Candida* species that typically originate from the host’s own microflora. Unlike many *Candida* spp., it is not among the commensal organisms of the human gastrointestinal tract, and it has a particular affinity for the skin in areas such as the axilla and groin. *C. auris* can quickly colonize individuals following exposure, and invasive infections may occur within days to months. Colonization can persist for several months or even indefinitely, emphasizing the importance of identifying asymptomatic carriers [4,5]. These individuals are at risk of further health complications and can also transmit the infection to others or contaminate the environment [6]. Infections resulting from *C. auris* exhibit a clinical presentation characterized by fungemia; skin abscess meningitis wounds; and ear, bone, or burn-wound infections [6,7].

Genomic analysis has demonstrated that a significant number of genes in *C. auris* are related to central metabolism, which is consistent with other pathogenic *Candida* spp., thereby enabling it to adapt to diverse environments. *C. auris* exhibits similar virulence activities to those of *C. albicans*, including the secretion of enzymes, iron acquisition, tissue penetration, and cell wall remodeling, although these traits may vary among strains [8]. Research has revealed that *C. auris* is capable of evading immune responses as it receives less targeting by neutrophils than does *C. albicans* [9,10]. Additionally, *C. auris* forms biofilms and adheres to surfaces [11]. Isolates of *C. auris* may aggregate, which impedes disruption and thereby promotes survival in hospitals. However, non-aggregating strains exhibit higher pathogenicity. *C. auris*’ thermotolerance allows it to persist in the hospital environment [8].

*C. auris* demonstrates a relatively low virulence compared to other *Candida* spp., but it poses a significant threat due to its remarkable resistance to multiple antifungal agents. This resistance is a key factor in the high mortality rate associated with *C. auris* [12,13]. Biofilm formation enhances resistance by trapping antifungal drugs within the extracellular matrix. Specifically, the rich mannanoglucan polysaccharide-ride content of the matrix can sequester up to 70% of triazole antifungals [12]. Some research suggests that C. auris biofilms are resistant to antifungal agents, such as fluconazole, whereas planktonic forms are less resistant to antiseptics [14,15,16]. Gene expansions linked to drug resistance and multidrug efflux pumps have been identified as factors contributing to formidable resistance in *C. auris*. These include mutations in specific genes responsible for resistance to azoles and echinocandins, as well as the activity of efflux pumps such as the ATP-binding cassette and major facilitator superfamily, which contribute to its remarkable azole resistance [15].

Researchers have investigated the antifungal properties of natural substances, such as EOs, which have been utilized in traditional medicine for their antimicrobial characteristics. EOs are intricate blends of volatile compounds produced by plants, and their broad-spectrum bioactivity against bacteria, fungi, and viruses has garnered considerable interest. The mechanisms through which they exert their antifungal effects are often attributed to cell membrane disruption, inhibition of cell wall formation, dysfunction of fungal mitochondria, oxidative stress induction, interaction with membrane proteins, and biofilm inhibition. These mechanisms offer a promising approach for overcoming drug resistance in pathogens [17,18].

The existing scientific literature examines the antifungal properties of EOs against a range of *Candida* spp., although reports on *C. auris* are comparatively scarce. Furthermore, the variable chemical composition of EOs due to factors such as geography, season, and plant origin presents challenges for standardization and quality control that have not yet been adequately addressed. Moreover, there is a deficiency of clinical studies assessing the safety and efficacy of EOs against *C. auris*, which represents a significant gap in the translation of laboratory findings into clinical practice [19].

This review offers a comprehensive summary of the current research (2019–2024) on the antifungal mechanisms of EOs against *C. auris*. By integrating traditional knowledge with contemporary scientific discoveries, this study sheds light on the specific mechanisms by which EOs exert their antifungal properties, including disruption of cell membranes, inhibition of cell wall formation, and inhibition of biofilms. Understanding these mechanisms is crucial for the development of targeted therapeutic interventions. Furthermore, the review identifies areas for additional research, such as the need for standardization of EO composition and the conduct of clinical trials, underscoring the importance of future research and potential clinical applications.

**Figure 1:**
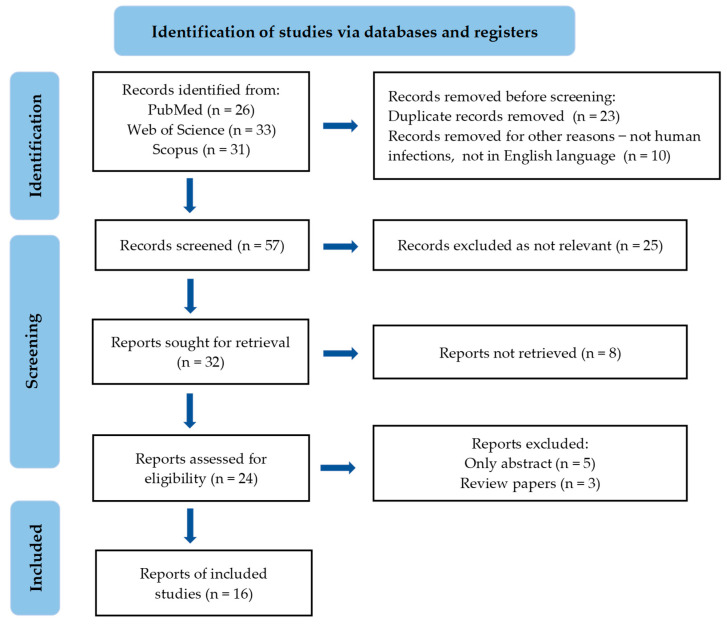
PRISMA flowchart of the included studies.

A comprehensive systematic literature review was conducted in accordance with the PRISMA methodology, encompassing studies published in English between 2019 and 2024. This search included PubMed, Scopus, and Web of Science, using keywords such as *C. auris*, EOs, and antifungal agents. The selection process comprised two stages. First, titles and abstracts were screened, followed by a full-text evaluation based on predefined inclusion and exclusion criteria. The inclusion criteria were studies published in English between 2019 and 2024. Exclusion criteria excluded studies that did not address the antifungal efficacy of EOs against human infection of *C. auris*, and those published before 2019 or in languages other than English. Data extraction involved collecting information from the authors, including the year of publication, study objectives, chemical composition, mechanisms of action, application areas, main findings, and conclusions. The extracted data were then synthesized and analyzed to provide a comprehensive overview. The results were categorized and presented based on the sources of EOs or compounds, mechanisms of action, and main results. No statistical analysis was performed, as this was a comprehensive literature review of scientific research. The PRISMA flow chart of the included studies is shown in Figure 1.

## 2. EOs and Their Chemical Constituents with Antifungal Activity—Mechanisms of Action

The antifungal effects of EOs and their chemical components are influenced by various mechanisms. They are composed of a diverse range of volatile compounds, such as terpenoids, aldehydes, and phenols, which exhibit extensive antimicrobial properties [17,18]. The ways in which EOs exert their antifungal effects are complex and include several primary mechanisms of action.

### 2.1. Disruption of Cell Membrane

EOs possess antifungal properties primarily through disrupting the integrity of cell membranes. Due to their lipophilic nature, EOs can blend with the lipid bilayers of fungal cell membranes, altering their structural and functional characteristics. Terpenoids, such as thymol, carvacrol, and eugenol, are key components in EOs that compromise the integrity of fungal cell membranes. These terpenoids contain a phenolic-OH group, which is critical for their antifungal activity. The presence of this group, with a delocalized electron system, facilitates proton exchange, resulting in membrane damage and disruption of ion homeostasis in fungal cells. The structural attributes vital to the antioxidant activity of terpenoids include the presence of a phenolic-OH group and hydrophobicity derived from the aromatic ring structure. These features are indispensable for disrupting cell membrane integrity and inducing changes that culminate in ion leakage and cell death. Additionally, the hydrophobic structure of the aromatic ring enhances penetration and damage to yeast plasma membranes, thereby augmenting their antifungal activity. The position of the-OH group within the molecule is also critical, as the phenolic-OH group attached directly to the benzene ring (forming phenol) is more effective in damaging membranes than an -OH group attached to the benzene ring via a methylene bridge [20]. Electron microscopy and transmission electron microscopy analyses were used to demonstrate that *Massoia aromatica* EO caused cell shrinkage, cytoplasmic leakage, and changes in cell shape of *C. albicans*, also increasing membrane permeability and leading to the leakage of cytosol and eventual rupture of the cell membrane [21].

### 2.2. Induction of Oxidative Stress

EOs can cause oxidative stress in fungal cells by producing reactive oxygen species (ROS). The accumulation of ROS can harm cellular components, such as lipids, proteins, and DNA, resulting in cellular dysfunction and death. Studies have shown that exposure of photogene to eugenol and citral increases intracellular ROS levels in a time-dependent manner, which is correlated with exposure time and contributes to the lethality observed in *C. albicans*. Cells exposed to eugenol at half the minimum inhibitory concentration (MIC) and hydrogen peroxide (H_2_O_2_) at all concentrations exhibited statistically significant ROS accumulation [22,23].

### 2.3. Mitochondrial Dysfunction

EOs exhibit an ability to target mitochondrial function, thereby impeding fungal energy metabolism. Specifically, compounds like thymol and carvacrol, which are present in EOs derived from thyme and oregano, have been demonstrated to disrupt the electron transport chain, thereby inhibiting the production of adenosine triphosphate (ATP) that is vital for the survival and proliferation of fungi. This mechanism not only restricts the energy generation of fungi, but also triggers programmed cell death pathways [22]. EOs possess the potential to hinder crucial enzymes that are involved in mitochondrial function, including those in the electron transport chain. By disrupting the functionality of these enzymes, they can impede ATP production and disrupt cellular respiration, ultimately resulting in mitochondrial dysfunction. Additionally, they can cause the permeabilization of mitochondrial membranes, leading to the release of pro-apoptotic factors like cytochrome c. This process activates apoptotic pathways in fungal cells, ultimately leading to cell death. Moreover, EOs can also alter mitochondrial metabolism by impacting processes such as oxidative phosphorylation and ATP synthesis. These alterations can disrupt the energy balance within fungal cells and contribute to their mitochondrial dysfunction [23].

### 2.4. Inhibition of Cell Wall Synthesis

EOs demonstrate antifungal properties by obstructing cell wall synthesis. The fungal cell wall, which primarily comprises glucans, chitin, and glycoproteins, serves as both structural support and protection. Certain components of EOs hinder the enzymes responsible for synthesizing these essential structural elements, jeopardizing cell wall integrity and culminating in fungal cell lysis. Additionally, EOs diminish ergosterol content, a vital component of the fungal cell membrane, leading to the disruption of membrane structure and functionality and ultimately resulting in cell death [24,25]. Eucalyptus EO has been shown to hinder the activity of ergosterol, which ultimately impairs membrane fluidity and performance [26]. The use of certain EO components has been found to inhibit the activity and expression of β-(1,3)-glucan synthase and chitin synthase, which are critical to the synthesis of fungal cell walls. This reduction in activity and the subsequent decrease in glucan and chitin synthesis leads to a weakening of the cell wall structure, ultimately resulting in their failure [24,26].

### 2.5. Interaction with Membrane Proteins

EOs not only have detrimental effects on cell membranes but can also impair the functioning of proteins associated with these membranes, such as ion channels, enzymes, and receptors. Membrane proteins that are vital for fungal survival can be adversely affected by EOs. By binding to specific sites on these proteins, EOs inhibit their activity, resulting in cellular dysfunction. EOs may cause conformational changes in membrane proteins, affecting their stability and operational capacity. These structural changes can impact the normal functioning of membrane proteins and the viability of fungal cells. Furthermore, EOs can inhibit the enzymatic activity of membrane proteins, which function as enzymes catalyzing important metabolic pathways within fungi. EOs have the potential to interfere with signal transduction pathways mediated by membrane proteins in fungal cells. By disrupting signaling mechanisms, EOs hinder intracellular communication, impairing fungal survival. Eugenol and citral have been shown to interact with amino acids in the cell membrane of *Penicillium roqueforti*, leading to changes in the conformation of membrane proteins that may contribute to the disruption of membrane function and integrity [26,27].

### 2.6. Biofilm Inhibition

**Figure 2:**
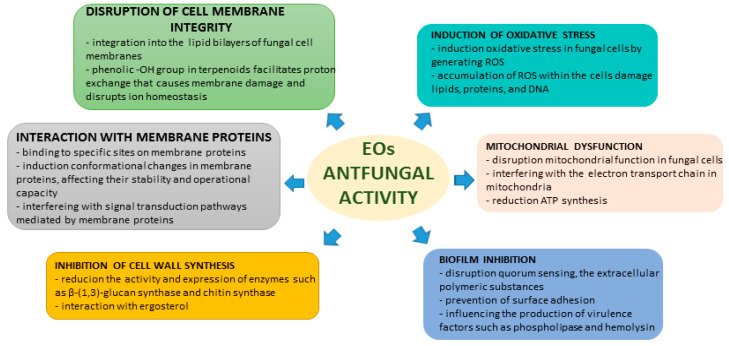
EOs’ mechanisms of antifungal activity.

Many pathogenic fungi often form biofilms, which are intricate communities encased within a matrix that exhibit heightened resistance to antifungal agents. Various studies have demonstrated that EOs can disrupt biofilm formation and eradicate previous pre-formed biofilms. For instance, thyme EO has been shown to inhibit biofilm formation by *Candida albicans* and to disrupt mature biofilms, presenting a promising therapeutic option for persistent fungal infections. EOs can disrupt quorum sensing, a critical communication process utilized by microorganisms to coordinate biofilm development. By interfering with quorum sensing, EOs hinder the ability of pathogens to form biofilms. EOs can also prevent *C. albicans* from adhering to surfaces, which is a vital step in biofilm formation. By blocking this initial attachment, EOs reduce the potential for its development. Additionally, EOs can influence the production of virulence factors, such as phospholipase and hemolysin, which are crucial for the establishment and maintenance of biofilms. By modulating these factors, they can weaken the structural integrity and defensive capabilities of biofilms. EOs have the potential to disrupt extracellular polymeric substances that maintain the structural integrity of biofilms, leading to their destabilization and potential eradication. Additionally, EOs often exhibit antimicrobial properties that can prevent and eliminate bacteria within biofilms, prevent further growth, and facilitate biofilm eradication. Certain compounds, such as limonene and eucalyptol, have been shown to inhibit biofilm formation in *Candida* spp. by targeting a critical factor in their pathogenicity [25,28,29]. The EO from *Massoia aromatic* was found to inhibit farnesol, a quorum-sensing molecule that is necessary for biofilm formation in *C. albicans*. This inhibition may play a role in the anti-biofilm activity of EOs [21]. A summary of the mechanisms of antifungal actions of EOs and their chemical constituents is provided in Figure 2.

## 3. EOs and Their Compounds against *C. auris*

**Figure 3:**
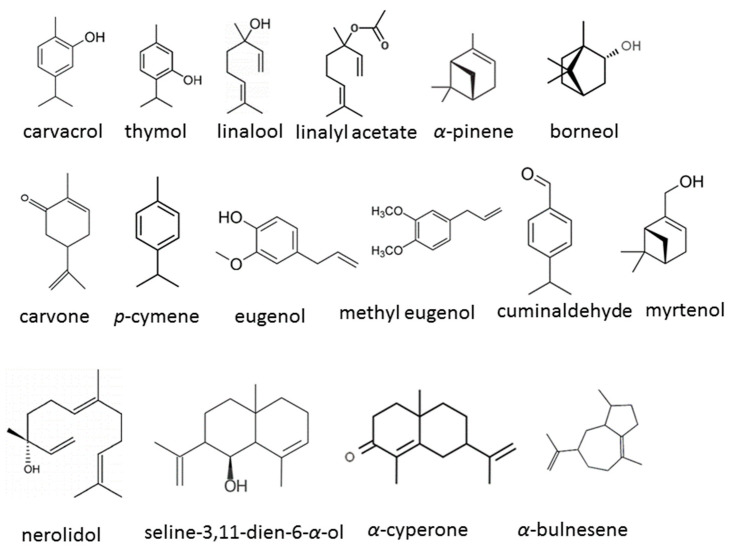
Chemical structures of the bioactive compounds identified in EOs in the studies.

A comprehensive analysis of 16 publications from 2019 to 2024 has been carried out to elucidate the diversity, efficacy, and mechanisms of action of EOs or their components in the management of *C. auris*. The chemical structures of some compounds identified in these studies are depicted in Figure 3, showcasing their chemical diversity. Additionally, Table 1 presents a brief overview of the publications assessed, encapsulating the breadth and findings of the research conducted thus far.

### 3.1. Diverse Sources of EOs and Their Compounds

Researchers have carried out extensive investigation into the antifungal properties of EOs obtained from a range of plant-based sources. [30,31,32,33,34,35,36,37,38,39,40,41,42]. These EOs encompass a wide array of bioactive compounds and exhibit potent antimicrobial activity against *C. auris*. Nevertheless, certain researchers have redirected their attention towards individual constituents or modified formulations to enhance antifungal properties or diminish the toxicity of particular compounds [43,44,45]. The following approach is a more efficient method of evaluation, as it allows precise assessment of the performance of individual EO components, rather than evaluating the EO as a whole, which may exhibit heterogeneity due to factors such as environmental or genetic variables (different chemotypes of the same species). By studying individual components or creating new formulations, such as liposomes, encapsulated EOs in nanostructured lipid carriers, or polycaprolactone-based nano-formulations, the researchers sought to optimize the therapeutic potential of these substances while minimizing the adverse effects of specific compounds [33,38,40]. The versatility and adaptability of plant products in the field of antifungal research are demonstrated by the diverse range of sources utilized by researchers, some of whom investigate whole EOs, whereas others examine individual compounds or employ modified formulations.

### 3.2. Methods of Antifungal Analysis

A multitude of techniques exist for assessing antifungal efficacy against *C. auris*. One widely employed method is the minimum inhibitory concentration (MIC) examination, which establishes the minimal concentration of EOs or compounds necessary to obstruct the growth of *C. auris*. This standardized process facilitates a quantitative evaluation of activity and offers valuable insights into the potency of the test substances. It was utilized in the majority of studies with the exception of four [30,33,37,44]. Various investigations have utilized the minimum fungicidal concentration (MFC) test, which aims to determine the minimum concentration of EOs or compounds that result in fungal cell death. This assessment extends beyond mere inhibition and examines the fungicidal activity of test substances, offering valuable insights into their capacity to eradicate populations of *C. auris* [31,34,38,39,41,43]. The evaluation of biofilm formation was carried out using biofilm formation assays to determine the influence of EOs or their compounds on *C. auris* biofilms. These assays are critical for devising effective strategies to combat fungal infections, as biofilms have significant impacts on microbial virulence and resistance [33,35,36,40,41,45]. One study utilized the disk-diffusion technique in conjunction with the paper-disc method to assess the efficacy of test substances against *C. auris*. This was achieved by evaluating the zone of inhibition surrounding the disks saturated with the substances in question, providing a rapid and reliable assessment of their efficacy against this organism [31,44]. *Lavandula angustifolia* EO-derived free and liposome-enveloped EOs have been investigated for their molecular mechanisms against *C. auris* using gene expression analysis in one study. This method allows researchers to determine the pathways through which natural products exert their effects by examining changes in gene expression in response to treatment [33]. Furthermore, many studies have been conducted to evaluate the potential synergistic effects between EOs and antifungal drugs. Researchers have found that the combination of these two agents can enhance the antifungal activity and reduce resistance development [34,39,40,45].

### 3.3. Proposed Mechanisms of EO Action against C. auris

Analyzing the processes associated with the interactions between EOs and *C. auris* is of paramount importance for evaluating their effectiveness. EOs possess the ability to disturb the cell membranes of *C. auris* due to their lipophilic nature, which may result in damage to the fungal cell membrane, disrupting its integrity and functionality [31,35]. The findings also demonstrated that the EOs caused damage to the cell membranes of *C. auris*, as indicated by the morphological changes observed such as the shrinking of cell surfaces and the receding of the cytoplasm, ultimately resulting in cell lysis. This damage compromised the integrity of the fungal cells and contributed to their demise. The degree of membrane damage observed in *Candida* cells was directly proportional to the concentration of cinnamon EOs used. Higher concentrations of EOs led to more severe damage to the cell membranes. This concentration-dependent effect suggests that EOs exert their antifungal activity by disrupting the structure and integrity of the fungal cell membrane. Cinnamon EOs contain bioactive compounds, including cinnamaldehyde and eugenol, which are known for their antimicrobial properties. These compounds possess lipophilic characteristics that enable them to penetrate the lipid bilayer of fungal cell membranes. This interaction can result in the disruption of the membrane structure, affecting its permeability and integrity. Ultimately, this disruption can lead to cell death and inhibit fungal growth. This phenomenon has been documented for other fungal pathogens as well [20,24]. EOs may exhibit antifungal properties by generating ROS, which trigger oxidative stress in fungal cells, resulting in their damage and in inhibition of their growth [33,36,45]. ROS are highly reactive molecules that can adversely affect various cellular components, such as proteins, lipids, and DNA. This oxidative stress response plays a critical role in suppressing fungal growth and biofilm formation [22]. Myrtenol, a compound isolated from plants in the genus *Taxus*, was observed to downregulate the expression of genes associated with biofilm formation in *C. auris* [45]. Specifically, the expression levels of essential genes involved in biofilm development were analyzed to elucidate the inhibitory effects of myrtenol. ERG11, a gene that plays a crucial role in ergosterol biosynthesis, was found to be downregulated by myrtenol. The disruption of ergosterol production caused by this downregulation can compromise the integrity of the fungal cell membrane, ultimately inhibiting biofilm formation. FKS1, which encodes the enzyme β-1,3-glucan synthase, is crucial for the synthesis of β-glucan, which is a key component of the fungal cell wall. By suppressing FKS1 expression, myrtenol is likely to hamper β-glucan synthesis, thereby impairing the structural integrity of the fungal cell wall and ultimately preventing biofilm formation. ALS5, a gene associated with surface adhesion, is a critical factor in the initial stages of biofilm development. The downregulation of ALS5 by myrtenol suggests that the compound disrupts *C. auris*’s ability to adhere to surfaces, thereby inhibiting the formation of biofilms [45]. The HOG1 gene, which participates in the oxidative stress-response pathway, exhibited variable expression patterns in response to treatment with *Lavandula angustifolia* EO. The study revealed an increase in HOG1 gene expression when liposome-encapsulated oil was used, suggesting activation of the oxidative stress-response pathway in *C. auris* biofilms. This upregulation may indicate a protective mechanism against the antifungal effects of encapsulated oil. Conversely, HOG1 was downregulated when free EO was applied, thereby potentially compromising the oxidative stress response in fungal cells. Concerning the CDR1 gene, which encodes an efflux pump, its expression changes in response to *L. angustifolia* EO treatment were also examined. Both the application of free *L. angustifolia* EO and of liposome-encapsulated oil upregulated the CDR1 gene. This alteration in gene expression may suggest a potential mechanism of resistance in *C. auris* biofilms treated with the EO, underscoring the need for further investigation into other genes associated with resistance [33]. This mechanism of action has also been observed in relation to cell wall synthesis, providing valuable insights into the molecular mechanisms underlying EOs’ antifungal activity [24,25,26]. EOs have demonstrated the ability to hinder the formation of biofilms in *C. auris* through several mechanisms of action, including the induction of oxidative stress [33,36], the downregulation of genes related to biofilm formation [33,45], and the interference or disruption of the extracellular fungal matrix that makes up the biofilm matrix [35,40]. These results align with the outcomes of other studies that have investigated the inhibitory impact of EOs on the biofilms of various pathogens [13,16,17,25,28,29]. Not all studies have specified the mechanism of action, which means that the specific ways in which the test substances affected *C. auris* were not clearly outlined or detailed [30,32,34,37,41,42,44]. The lack of specification regarding the mechanism of action suggests that the authors did not provide clear information on how the substances interact with the pathogen at the molecular or cellular level to inhibit its growth or virulence. This absence of a specific mechanism of action in these studies may hinder the in-depth understanding and characterization of the antimicrobial properties of the substances studied. It can also be inferred that these studies primarily focused on demonstrating the general antimicrobial activity of the substances, rather than on elucidating the exact biological pathways or targets through which they exert their effects on *C. auris*. In scientific research and the scientific literature, determining the mechanism of action is essential to providing insight into how antimicrobials operate, understanding their interactions with pathogens, and identifying new targets for therapeutic intervention. When the mechanism of action is not clearly defined, it can lead to gaps in understanding of how substances function against pathogens and restrict the ability to optimize efficacy or develop targeted antimicrobial strategies.

### 3.4. Findings

The studies conducted thus far have consistently indicated substantial antifungal effects against *C. auris* of the substances under investigation, with MIC and MFC values exhibiting diversity, which may be attributed to the distinct chemical positions of the analyzed EOs. Moreover, some studies have revealed the potential of the tested substances to affect *C. auris* biofilm formation [35,45]. The results showed a reduction in biofilm mass and interference with biofilm structures, highlighting the potential of EOs to interfere with virulence factors associated with pathogen biofilms. Synergistic interactions between the test substances and conventional antimicrobial agents were also studied [34,39,43,45]. These results indicated synergistic effects when the substances were combined with antifungal drugs, potentially increasing their effectiveness in inhibiting *C. auris* biofilms and overcoming drug resistance. The effects of these substances on gene expression in *C. auris* were investigated, revealing downregulation of genes related to fungal cell wall synthesis and upregulation of stress-response genes, providing insights into the molecular mechanisms underlying antimicrobial activity [33,45]. Some studies demonstrated an increase in ROS production in *C. auris* cells exposed to the test substances [33,45]. This oxidative stress response played a key role in the inhibition of fungal growth and biofilm formation, highlighting a key mechanism of action against pathogens. The effects of these substances on virulence factors of *C. auris* were also evaluated, which demonstrated inhibition of hemolytic activity and adhesion capacity, thus suggesting a potential role in reducing virulence and pathogenicity of the pathogen [31,43]. Considering the diverse mechanisms through which EOs and compounds exert their antimicrobial activity against *C. auris*, these findings underscore the potential of these natural products as promising agents for combating fungal infections.

## 4. Limitations, Challenges, and Future Directions

Although the antifungal properties of EOs have been documented against *C. auris*, there are still significant limitations to current findings. These studies were conducted in vitro, under conditions that do not always accurately mimic the complex biological environments found in vivo. The chemical complexity of EOs, which can contain hundreds of bioactive compounds, complicates the identification of the active ingredients and their mechanisms of action. This variability makes it difficult to standardize treatments and accurately predict therapeutic outcomes. Translating the in vitro antifungal efficacy of EOs into clinical applications faces several obstacles. First, their safety profiles must be carefully evaluated, as some compounds can be toxic or cause allergic reactions. The volatility and instability of EOs present additional challenges for their formulation and delivery in the clinical setting. Achieving effective concentrations without harming the host is a major challenge. As with all antimicrobial agents, the development of resistance to EOs poses a serious threat. Although they are thought to have a lower risk of inducing resistance owing to the complex mixtures of compounds that act on multiple microbial targets, there may still be cases of reduced susceptibility.

To overcome these challenges and unlock the potential of EOs for antifungal applications, a number of future research avenues can be proposed. The creation of standardized EO formulations calls for the use of advanced analytical techniques to identify and characterize their bioactive components. By doing so, stable, bioavailable, and effective products can be formulated. Cutting-edge technologies such as nanoparticles, liposomes, and emulsions can be employed to demonstrate the delivery and targeting of EOs, thus enhancing their efficacy and safety. Combining EOs with conventional antifungal drugs or other antimicrobial agents can improve their effectiveness and reduce the likelihood of resistance. High-throughput screening of EO components using existing antifungals can be employed to identify synergistic combinations. Undertaking rigorous clinical trials is necessary to confirm the efficacy and safety of EO-based therapies. Conducting comparative studies on the use of EOs and conventional antifungal therapies is crucial to establishing the therapeutic potential of these treatments.

## 5. Conclusions

This comprehensive review presents recent studies that have explored the impact of EOs and their constituents on *C. auris*, a multidrug-resistant fungal pathogen. The results of these studies indicate that the EOs and their compounds that were tested exhibited potent antifungal activity against *C. auris*, as evidenced by the various MIC values recorded. These compounds were found to inhibit biofilm formation and disrupt biofilm structure, which is crucial for the pathogen’s virulence and persistence. Moreover, their combination with antifungal drugs resulted in synergistic effects that enhanced their efficacy in inhibiting *C. auris* and potentially overcoming drug resistance. The compounds also modulated gene expression in *C. auris*, leading to the downregulation of biofilm-related genes and the upregulation of stress-response genes. The induction of ROS in *C. auris* cells by the EOs is a key factor in inhibiting the growth of this fungus. Additionally, they showed inhibitory effects on *C. auris* virulence factors such as hemolytic activity and adhesion capacity. The results of these studies suggest the promising potential of EOs and compounds against *C. auris*. However, significant challenges must be addressed in order to realize their full potential in clinical settings. These challenges include variability in chemical composition, concerns about toxicity and allergenicity, stability issues, and the lack of standardized dosing regimens. Furthermore, the mechanisms of action must be clearly defined to facilitate the development of more effective and safer therapeutic forms. Future research endeavors should concentrate on overcoming the challenges associated with EOs by expanding their chemical characterization and improving delivery systems to enhance bioavailability and reduce toxicity. Additionally, controlled clinical trials should be conducted to establish efficacy and safety profiles. Research should also focus on understanding the mechanisms underlying potential resistance to EOs and developing strategies to mitigate this risk. EOs and their constituents possess significant potential for the treatment of fungal infections, and their therapeutic potential can be expanded through multicenter research and interdisciplinary collaboration. In addition to scientific and technological advancements, a regulatory framework is necessary to support the safe, effective, and sustainable use of EOs in clinical settings.

## Figures and Tables

**Table 1:** Summary of the review of the evaluated studies.

References	Source of Essential Oil/Compound	Methods of Analysis	Mechanisms of Action against *C. auris*	Results
[30]	*Thuja plicata*	Spectrophotometric method to estimate population growth parameters, including carrying capacity (K) and intrinsic growth rate (r) of *C. auris*. under varying doses of EOs.	N/A	The study showed that EO had a marginally significant inhibitory effect on the transmissibility (K) of *C. auris*, as indicated by the results of one-way ANOVA. Paired post-hoc tests with adjustment for multiple comparisons showed no significant differences between dose pairs for K. EO inhibited the intrinsic growth rate (r) of *C. auris*, as indicated by the results of one-way ANOVA. These results suggest that EO affected the growth parameters of *C. auris* populations, including K and r.
[31]	*Cinnamomum zeylanicum* bark and leaf	Disc diffusion (direct and vapor) assay. MIC determination. MFC determination. Morphological interference assays. Haemolysin production assay.	EOs reduce the hemolytic activity of *C. auris*, which is important in preventing host cell damage during infection. Morphological interference tests showed damage to the cell membrane of *C. auris*, indicating a potential mechanism of action of EOs. Both EOs from leaves and cinnamon bark inhibited the formation of filaments in *C. auris*, which is crucial for its pathogenicity and invasive properties.	Direct and vapor-diffusion assays showed greater inhibitory activity of bark EO compared to leaf EO against *C. auris*. MICs and MFCs of bark essential oil were below 0.03% (*v*/*v*), which was lower than the MICs of leaf EO (0.06–0.13%, *v*/*v*). The MFCs of bark EO against *C. auris* strains were at 0.25% (*v*/*v*). Morphological interference assays demonstrated damage to the cell membrane of *C. auris* by both cinnamon leaf and bark EOs, and inhibition of hyphae formation. The hemolysin production assay showed that the EOs could reduce hemolytic activity.
[32]	Structural modifications of natural cuminaldehyde isolated from *Calligonum comosum*	MIC determination.	N/A	MIC50 of modified compounds range from 2 to 15 μg/mL, whereas for amphotericin B it was 0.3 μg/mL.
[33]	*Lavandula angustifolia* (linalyl acetate, linalool) free and liposome-enveloped EOs	Biofilm formation and treatment. ROS production and gene expression. Microbiological assays of free and liposome-encapsulated *L. angustifolia* EOs. Confocal laser scanning microscopy—biofilms were treated with caspofungin, stained with specific dyes, and observed under a microscope to assess the impact of the essential oil on biofilm formation and cell viability.	Membrane disruption by EOs, due to their lipophilic nature, can penetrate the cytoplasmic membrane of fungal cells, leading to membrane damage. Accumulation of intracellular ROS. EO was found to modulate the expression of these genes, with downregulation of ERG11 and ALS5, and upregulation of HOG1 and CDR1. Showed synergistic action of EOs’ major components, such as linalyl acetate and linalool.	The significant effects of free and liposome-enveloped EOs on the planktonic growth of *C. auris* were observed even at a concentration of 0.01% (*v*/*v*). Significant increase observed in intracellular ROS in biofilm after application of EOs at 0.5% (*v*/*v*).
[34]	*Melaleuca alternifoli*, *Pelargonium graveolens*, *Citrus aurantifolia* peel, *Eucalyptus* spp., *Mentha piperita*, *Leptospermum scoparium*, *Syzygium aromaticum* bud, *Commiphora abyssinica*, *Mentha spicata*, *Cinnamomum zeylanicum*, *Citrus bergamium*, *Citrus limon*, *Boswellia* spp., *Coriandrum sativum*, *Citrus × aurantium* subsp. *amara*, *Citrus × aurantium* var. *paradisi*, *Lavandula* spp., *Zingiber officinale*, *Ocimum basilicum*, and *Cymbopogon schoenanthus*	MIC determination. MFC determination. Synergism testing interactions between essential oils and antifungal drugs.	N/A	MIC by percentage (*v*/*v*) from 0.01% to 0.5%. MFC by percentage (*v*/*v*) from 0.02% to 0.5%. Interactions between select antifungal drugs and EOs: micafungin-cinnamon bark and clove bud not significant, lemongrass additive, flucytosine-cinnamon bark additive, clove bud synergistic, lemongrass additive; amphotericin B-cinnamon bark not significant, clove bud antagonistic, lemongrass additive; fluconazole-cinnamon bark not significant, clove bud synergistic, lemongrass additive.
[35]	*Melaleuca leucadendra*, *Melaleuca viridiflora*, *Melaleuca alternifolia*, and *Thymus zygis*	MIC determination. Biofilm formation assay and assessment of pre-formed biofilms.	Essential oils have been shown to damage the membranous structures of fungal cells, leading to membrane disruption and permeabilization. Components of essential oils, such as carvacrol, terpinen-4-ol, and α-terpineol, can modify membrane permeability by interacting with membrane proteins, affecting membrane fluidity and structure.	MIC50 values ranged between 0.78% and 1.56; *M. alternifolia* EO exhibited the lowest MIC50 value of 0.78%. Direct application of EOs at 2.4% (*v*/*v*) led to a total inhibition of biofilm growth; *T. zygis* and *M. leucadendra* EOs induced a total eradication of 24-h-old biofilms; *M. alternifolia* and *M. viridiflora* EOs significantly reduced viable cells in pre-formed biofilms, with reductions of 5 Log10 CFU/mL and 4 Log10 CFU/mL, respectively.
[36]	*Juniperus oxycedrus* ssp. *macrocarpa* (α-pinene)	MIC determination. Disk-diffusion method. Biofilm production assessment.	The antifungal effect of the EO may be attributed to reactive oxygen species production and alteration of expression of biofilm-related genes. The lipophilic property of the oil enhances penetration of hydrophobic compounds into the cytoplasmic membrane, leading to membrane damage.	MIC value against planktonic cells of *C. auris* was found to be 0.02 (*v*/*v*%); MIC value against one-day-old biofilms of *C. auris* was 1.56 (*v*/*v*%). Median FICI was 0.088, indicating strong fungicidal activity.
[37]	*Thymus vulgaris* (thymol 63.1%), *Thymus zygis* (thymol 26.5%), *Thymus satureioides* (borneol 29.3%), and *Thymus mastichina* (linalool 31.9%)	Cell viability. determination by counting CFU and presented as CFU per milliliter (Log10 (CFU/mL)). Agar disk-diffusion assay.	N/A	*T. vulgaris* EO showed the most significant antifungal effect with a halo of 59.75 ± 15.75 mm. *T. mastichina* EO presented a halo of 13.13 ± 1.36 mm, indicating lower antifungal activity. *T. zygis* and *T. satureioides* EO showed varying levels of antifungal activity, with different chemical profiles compared to *T. vulgaris*.
[38]	*Lippia sidoides* (thymol 68.22%), encapsulated EO in nanostructured lipid carrier	Agar diffusion test. MIC determination. MFC determination.	High rates of thymol retention in the range of 91% to 100% in the nanostructured lipid carrier loaded with *L. sidoides* EO. It is attributed to the lipophilic nature of the EO, which facilitates greater partitioning of thymol into the lipid matrix and less into the aqueous phase.	The MIC values for EO and EO-loaded nanostructured lipid carrier ranged between 0.281 and 0.563 mg/mL, and MFC–0.140 and 0.562 mg/mL.
[39]	*Cymbopogon martini*, *Cymbopogon citratus*, *Elettaria cardamomum*, *Coriandrum sativum*, *Anethum graveolens*, *Helichrysum italicum*, *Cuminum cyminum*, *Mentha piperita*, *Melaleuca alternifolia*, *Rosmarinus officinalis*, *Thymus vulgaris* with thymol chemotype *Cinnamomum zeylanicum* (bark), *Pelargonium graveolens*, *Cinnamomum camphora*, and *Lavandula angustifolia*	MIC90 determination. MFC90 determination. Checkerboard tests were conducted to investigate the synergy between *C. zeylanicum* EO, its active fraction and cinnamaldehyde (the main chemical compound) with fluconazole. *C. zeylanicum* EO toxicity was evaluated in *Galleria mellonella* larvae to assess its safety at different concentrations.	*C. zeylanicum* EO, used in low concentrations, likely inhibits ATPase pump activity in *C. auris* cells. *C. zeylanicum* EO, and its active fraction act synergistically with fluconazole, increasing the antifungal activity of the drug.	EO from *C. zeylanicum* was the most active of the 15 EOs tested, with an MIC90 of 0.06% by volume and an MFC90 of 0.06% by volume. Cinnamaldehyde, as the main component of EO from *C. zeylanicum,* showed antifungal activity, suggesting its role in efficacy against *C. auris*. Co-presence of EO from *C. zeylanicum* or its active fraction with fluconazole at therapeutic concentrations improved the drug’s efficacy against *C. auris*. However, cinnamaldehyde alone showed additive rather than synergistic effects with fluconazole.
[40]	*Cinnamomum cassia* and its nano-formulations based on polycaprolactone	Micro-broth dilution tests. Checkerboard tests to evaluate the synergistic action with antifungal. agents like micafungin or fluconazole. Biofilm disrupting activity evaluation.	Cinnamaldehyde exerts its antifungal action by inhibiting ATPases. Inhibition the biosynthesis of the fungal cell wall by cinnamaldehyde. Alteration of the fungal membrane structure and integrity by cinnamaldehyde.	The average MIC values are lower in the nanoformula than in the pure compound (0.01 and 0.04, respectively). Tests conducted with the empty nanoformula confirm their antifungal ineffectiveness.
[41]	*Lippia alba* (carvone-limonene chemotype) and *L. origanoides* (thymol chemotype), *L. micromera* (*p*-cymene chemotype)	MIC and MFC determination. Biofilm formation were determined using a crystal violet assay in 96-well round-bottom microplates incubated for 48 h at 35 °C.	N/A	MIC values from 188 to 750 μg/mL, MFC values from 375 to 563 μg/mL. MBIC50 values from 53 to 141 μg/mL. Compounds like thymol and *p*-cymene showed a negative correlation with antifungal activity, suggesting that higher concentrations of these compounds may result in better antimicrobial activity.
[42]	*Lippia origanoides* different chemotypes	MIC determination. Cytotoxicity testing on the HaCaT cell line.	N/A	MIC values for single EO compounds were as follows: Limonene, had the highest activity against *C. auris*, with a MIC range of 16–64 μg/mL; thymol had MIC values ranging from 16 to 128 μg/mL; carvacrol had MIC values ranging from 32 to 128 μg/mL *p*-cymene had MIC values ranging from 181 to 256 μg/mL. MIC values for EOs of *L. origanoides* (thymol chemotype) from 16 to 64 μg/mL, and for *L. origanoides* (*p*-cymene chemotype) from 181 to 256 μg/mL. Thymol had CC50 of 427.5 μg/mL, SI range of 3.3–6.7; p-Cymene had CC50 of 831.2 μg/mL, SI range of 3.2–5.5; Carvacrol had CC50 of 410.7 μg/mL, SI range of 1.6–12.8; Limonene had CC50 of 400.5 μg/mL, SI range of 3.1–50; EOs form *L. origanoides* (thymol chemotype)—CC50 value of 903.6 μg/mL, *L. origanoides* (carvacrol-thymol chemotype)—CC50 value of 788.0 μg/mL, *L. origanoides* (carvacrol-*p*-cymene chemotype0 a CC50 value of 877.9 μg/mL, *L. origanoides* (thymol-*p*-cymene chemotype)-CC50 value of 665.9 μg/mL.
[43]	carvacrol, thymol, eugenol, methyl eugenol	MIC determination. MFC determination. Combination studies with fluconazole, amphotericin B, caspofungin, and nystatin. Evaluation of virulence factors.	Inhibition of *C. auris* growth. Inhibition of adherence to epithelial cells and reduction of proteinase production.	Carvacrol exhibited the best MIC value of 125 μg/mL, followed by thymol with an MIC of 312 μg/mL. MFC values were as follows: carvacrol, 250 μg/mL; thymol, 1250 μg/mL; eugenol, 2500 μg/mL; methyl eugenol, ≥2500 μg/mL. Combination of carvacrol with fluconazole, amphotericin B, nystatin, and caspofungin resulted in synergistic and additive effects in 68%, 64%, 96%, and 28%, respectively.
[44]	α-cyperone	Bioscreen-C growth monitoring system. Paper discs containing α-cyperone (60 μg/disc).	N/A	α-Cyperone inhibited the growth of *C. auris* at the concentrations used.
[45]	myrtenol	MIC determination. Biofilm formation at sub-MICs using a 96-well microtiter plate and techniques such as crystal violet staining, XTT reduction assay, and CFU counts. Synergistic interaction between myrtenol and antimicrobial drugs using the checkerboard method Expression of specific genes associated with bacterial motility, adhesion, and biofilm formation. Measuring ROS production that plays a role in inhibiting biofilm formation.	Downregulation of genes related to biofilm formation, including ERG11 and FKS1 involved in fungal cell wall synthesis, and ALS5 associated with adherence to substrates. Induction of oxidative stress leading to an increase in intracellular and mitochondrial ROS production in *C. auris* biofilms. Strong synergistic effects when combined with antifungal drug caspofungin and antibiotic meropenem in inhibiting the growth of *C. auris* biofilms.	MIC between 6.2 and 50 μg/mL when tested against *C. auris* single and mixed biofilms Myrtenol exhibited significant antibiofilm activity. At a concentration of 12.5 μg/mL, a reduction in biofilm mass was observed compared to the untreated control. Myrtenol showed strong synergistic effects when combined with caspofungin (CAS) and meropenem (MEM). The most effective combinations were observed at the following concentrations: 2.5 μg/mL myrtenol, 0.01 μg/mL CAS for an 80% biofilm inhibition; 12.5 μg/mL Myrtenol, 0.1 μg/mL MEM. Myrtenol tested at a sub-MIC concentration of 12.5 μg/mL, led to an increase in intracellular reactive oxygen species (iROS) and mitochondrial reactive oxygen species (mROS) in response to its exposure. Measurements showed a sixfold increase in iROS and an eightfold increase in mROS compared to untreated biofilms.

N/A—not assessed; MIC50—minimum inhibitory concentration for 50% inhibition; MFC—minimum fungicidal concentration; MBIC50—minimum biofilm inhibitory concentration; HaCaT—cell line derived from non-tumor keratinocytes; CC50—50% cytotoxic concentration; SI—selectivity index; ROS—reactive oxygen species; FICI—fractional inhibitory concentration index; MIC90—minimum inhibitory concentration at 90% inhibition; MFC90—minimum fungicidal concentration at 90% kill; CFU—colony-forming units; BMIC—biofilm minimal inhibitory concentration in testing.

## Data Availability

No new data were created.
